# Editorial: Advances in brain-computer interface technologies for closed-loop neuromodulation

**DOI:** 10.3389/fnins.2023.1327533

**Published:** 2023-11-10

**Authors:** Yuchen Xu, Hubin Zhao, Cosimo Ieracitano

**Affiliations:** ^1^Department of Bioengineering, University of California, San Diego, San Diego, CA, United States; ^2^Division of Surgery and Interventional Science, University College London, London, United Kingdom; ^3^Dipartimento di Ingegneria Civile, dell'Energia, dell'Ambiente e dei Materiali (DICEAM), University Mediterranea of Reggio Calabria, Reggio Calabria, Italy

**Keywords:** brain-computer interface (BCI), neuromodulation, fNIR, EEG, sensors

## Introduction

Both the diagnosis and treatment of neurodegenerative disorders have historically presented significant challenges. The complexity of diseases such as dementia due to Alzheimer's disease or epilepsy frequently exceed the effectiveness of current therapeutic interventions. However, therapeutic neuromodulation through the use of brain-computer interfaces (BCI) and neurofeedback (NF) technologies shines as a promising beacon of transformation.

The concept of a closed-loop brain interface refers to the seamless integration of neural recording, decoding, and stimulation. This harmonious integration aims not only to bridge gaps, but also to foster collaboration among diverse scientific disciplines. electrophysiological techniques such as electrocorticography (ECoG) and non-invasive electroencephalography (EEG) have paved the way for BCIs. Nevertheless, with the continuous advancement of science and technology, the methods and approaches within the BCIs also progress.

This Research Topic focuses on emerging modalities and methodologies regarding BCI and NF technologies. The dawn of innovative neural recording techniques has broadened the horizons of BCIs. The emergence of in-ear EEG, functional near-infrared spectroscopy (fNIRS), and functional magnetic resonance imaging (fMRI), is accompanied by a rise in wearability, improved spatial resolution, and enhanced temporal resolution. Such advancements not only refine data quality but also enhance patient comfort and compliance. Neural decoding, the intricate art of deciphering the brain's complex signals, has also witnessed groundbreaking advancements. Beyond the realms of traditional feature extraction methodologies such as Support Vector Machine (SVM), Independent Component Analysis (ICA), and Common Spatial Pattern (CSP), newer approaches based on deep learning and transfer learning hold the potential to enhance the depth and precision of neural interpretations. Such evolutions signify a transition from merely understanding brain signals to anticipating and leveraging them for therapeutic interventions. On the forefront of neurostimulation, diversification beyond electrical stimuli heralds a new era. The integration of acoustic and optical stimuli, evidenced in applications like cochlear implants, underscores the potential of multi-modal neurostimulation in enhancing therapeutic outcomes.

## Highlights of this Research Topic

On the neural recording aspect, Li R. et al. introduce an impactful innovation—a high sampling rate and multichannel wireless recorder. This innovation transcends the challenges of EEG miniaturization, scalability, and portability, laying the foundation for brain activity monitoring in BCIs.

On the neural recording aspect, deciphering the complex neural lexicon is integral for BCI effectiveness. Sun and Mou delve deep into EEG-based signal processing, presenting a comprehensive review that ranges from foundational preprocessing to the avant-garde realms of deep learning in EEG signal decipherment. Their synthesis offers insights into both the achievements and tribulations in EEG categorization, illuminating the trajectory for future explorations. Zhao et al. spotlight the learning-based motion artifacts processing in fNIRS, emphasizing the imperativeness of negating motion-induced disturbances for the accuracy of neurovascular studies.

On the neurostimulation aspect, Li M. et al. study on the stimulation enhancement effect when exoskeleton-assisted hand rehabilitation is harmoniously merged with fingertip haptic stimulation. This study stands as a testament to the potential of amalgamating neuro-stimulatory approaches, especially in the domain of stroke rehabilitation. In another work, Guo et al. introduce us to the pioneering “Temporally interfering electrical stimulation”—a non-invasive brain stimulation technique that beckons a paradigm shift by targeting specific deep brain regions.

## Future directions and summary

The field of BCIs is poised for a revolutionary transformation. With the increasing adoption of innovations in wearable devices, implantable systems, and cutting-edge neural interfaces, the potential of BCIs extends beyond clinical interventions. Whether it involves creating new circuits, sensors, actuators, or the exploration of machine learning and innovative neurophysiological feedback approaches, each step forward strengthens the foundation of BCI technology.

This Research Topic is a testament to the collective passion and determination of the global scientific community. Our aim remains unwavering—to champion the cause of BCIs, ensuring their transition from specialized scientific domains to universally accessible solutions, addressing a spectrum of neurological challenges.

## Author contributions

YX: Writing—original draft, Writing—review & editing. HZ: Writing—review & editing. CI: Writing—review & editing.

